# Effect of Biomedical Hydrogels on Exercise-induced Muscle Damage: A Narrative Review

**DOI:** 10.2174/0109298673360294241217061953

**Published:** 2024-12-26

**Authors:** Jie Cai, Mingbo Fan, Ailin Yu, Chenghu Wu

**Affiliations:** 1 Department of Orthopaedics, The First People's Hospital of Xiaoshan District, Xiaoshan Affiliated Hospital of Wenzhou Medical University, 311200, Zhejiang, China;; 2 Department of Neurosurgrey, The Central Hospital of Wuhan, Tongji Medical College, Huazhong University of Science and Technology, Wuhan, 430014, China;; 3 Department of Endocrinology, Renmin Hospital of Wuhan University, Wuhan, 430060, China;; 4 Department of Biomedical Engineering, Wenzhou Medical University, 325035, Wenzhou, China;

**Keywords:** Exercise-induced muscle damage, biomedical hydrogels, prevention and treatment, drug release systems, hydrophilic polymers, physical therapy

## Abstract

Exercise-induced muscle damage (EIMD) is a common occurrence among athletes and individuals engaged in physical fitness activities. Muscle strains result from excessive or repetitive muscle tension, leading to tissue damage, inflammation, and pain. These strains can range from mild discomfort to severe damage, resulting in pain, inflammation, and reduced functionality. Effective management of muscle damage is crucial for promoting recovery and returning individuals to their desired level of activity. Conventional treatment modalities such as rest, ice, compression, and elevation (RICE), physical therapy, and nonsteroidal anti-inflammatory drugs (NSAIDs) have limitations in terms of efficacy and long-term outcomes. Consequently, there is a need for innovative approaches that not only address the symptoms but also promote healing and prevention of future injuries. Hydrogels are three-dimensional crosslinked networks of hydrophilic polymers that have gained significant attention in the field of biomedicine. Their unique properties, drug-delivery capabilities, and capacity to provide mechanical support make them promising tools in muscle damage management. Biomedical hydrogels hold significant potential as a preventive or alleviative approach for EIMD. This review provides a comprehensive overview of biomedical hydrogels as a promising approach for preventing and alleviating EIMD, addressing current challenges, and outlining future directions for research and development in the field.

## 1 INTRODUCTION

**Fig. (1):**
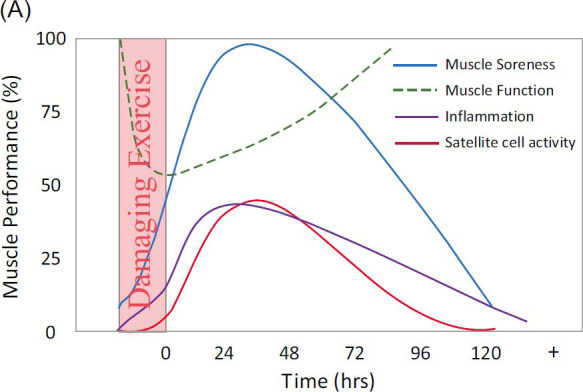
Time course of events following a bout of muscle damaging exercise (copyright acquired from Owens *et al.* [3]).

Exercise-induced muscle damage manifests as muscle soreness or discomfort alongside a notable reduction in muscle strength within the initial 12-72 hours following exercise [1]. Additionally, EIMD triggers an inflammatory response marked by leukocyte activation, muscle edema, compromised muscle function, delayed-onset muscle soreness (DOMS), and a series of intracellular processes aimed at restoring affected muscle integrity [2]. EIMD is typified by symptoms persisting from immediately post-exercise to approximately 14 days thereafter [3, 4]. Athletically, its primary repercussion lies in the diminishment of skeletal muscle function and the experience of soreness [1, 5]. These symptoms adversely affect functional capacity, muscle soreness, and exercise capacity, and disrupt the perception of force production and limb positioning [6]. The severity and duration of these manifestations, and consequently their impact on performance, are contingent upon the intensity and duration of the provoking exercise as well as individual susceptibility to such stimuli [7]. Given their potential to impair performance, the declines in muscle function and increases in muscle soreness associated with EIMD are of considerable significance to athletes (Fig. **1**) [3, 4, 8] .

High-force eccentric muscle actions commonly induce ultrastructural muscular disruption, DOMS, elevation in specific intramuscular proteins in circulation, swelling of the affected limb, decreased range of motion, and impaired muscle force-producing capacity [2, 9, 10]. Exercises prone to eliciting these symptoms include resistance training, prolonged running, downhill running, and intermittent, high-intensity exercise [10]. The severity of damage resulting from eccentric actions is heightened when executed at extended muscle lengths, with increased forces, and at accelerated angular velocities [8, 11]. Muscle susceptibility to damage may be mitigated during subsequent bouts following prior exposure to eccentric exercise, a phenomenon known as the repeated bout effect (RBE) [12]. The assessment of muscle damage extent typically involves measuring various indirect markers, with reduced muscle force post-eccentric exercise being the most suitable indicator [13, 14]. Depending on the aforementioned factors, post-exercise force losses range between 15% and 60% of predamage values and may persist for approximately two weeks [14, 15].

The intricate mechanisms underlying EIMD primarily stem from physical trauma to the sarcomere and sarcolemma induced by eccentric lengthening and excitation-contraction (E-C) coupling failure [16]. Despite being commonly assessed, the precise etiology of DOMS remains elusive [17, 18]. The sensation of muscle soreness may arise from a multifaceted interplay of structural muscle damage, disrupted calcium (Ca^2+^) homeostasis, and sensitization of nociceptors by inflammatory cell infiltration [18, 19]. However, findings indicating heightened muscle soreness following eccentric exercise in the absence of significant inflammation challenge the conventional understanding of DOMS. DOMS typically manifests between 8 and 24 hours post-exercise, peaks between 24 and 48 hours, and typically resolves within 96 hours [20]. Furthermore, the elevation of muscle-specific proteins such as muscle-specific creatine kinase (CK) in plasma and myoglobin in serum, peaking 2-6 days following the initial insult, is commonly observed [21]. The eccentric lengthening-induced membrane damage results in augmented membrane permeability, leading to the leakage of muscle proteins into circulation, particularly in the immediate aftermath of EIMD [3, 8, 22]. However, circulating muscle-specific proteins exhibit a poor temporal correlation with muscle function and are thus better utilized as indicators of tissue damage occurrence rather than for assessing its magnitude [23]. The intricate mechanisms associated with EIMD can be conceptualized into two distinct phases: (i) the primary damage phase, arising directly from the mechanical work exerted, and (ii) the secondary damage phase, characterized by the amplification of tissue damage through processes intertwined with the inflammatory response [24-26].

**Fig. (2):**
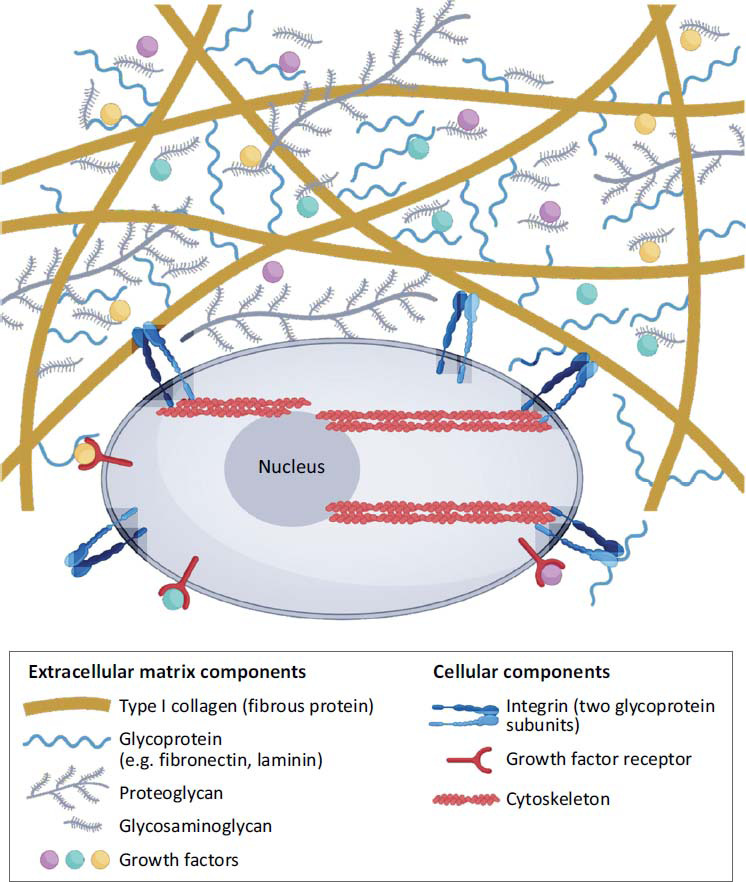
Chemical strategies to engineer hydrogels for cell culture. The cellular microenvironment in tissues. (copyright acquired from Lou *et al.* [31]).

Biomedical hydrogels present a multitude of properties and advantages, positioning them as promising contenders for the management of EIMD [27-29]. Constituting three-dimensional networks of hydrophilic polymers, hydrogels possess the capability to absorb and retain significant volumes of water. Notably, their high biocompatibility akin to the natural extracellular matrix (ECM) environment facilitates interactions with cells and integration with tissue (Fig. **2**) [30-32]. The viscoelastic attributes of hydrogels confer mechanical support to injured muscle tissues, thereby mitigating strain and forestalling further damage. Moreover, hydrogels can be tailored to administer therapeutics directly to the afflicted area, affording controlled release of drugs for localized pain alleviation, inflammation mitigation, and tissue regeneration [33, 34]. An understanding of the underlying mechanisms governing the utilization of biomedical hydrogels in muscle strain management is imperative.

Primarily, their augmented biomechanical reinforcement aids in dispersing mechanical burdens, mitigating stress concentration on injured muscle fibers. This mechanical fortification fosters proper alignment of collagen fibers during the healing trajectory, facilitating tissue repair and averting excessive scar formation. Secondly, hydrogels can be impregnated with analgesics, anti-inflammatory agents, growth factors, or stem cells, which are dispensed in a regulated manner to modulate the local microenvironment and bolster tissue regeneration [35, 36]. This targeted delivery paradigm enhances therapeutic efficacy while curbing systemic side effects.

A plethora of studies have delved into the potential of biomedical hydrogels for managing EIMD. Preclinical investigations employing animal models have elucidated the efficacy of hydrogels in attenuating inflammation, fostering muscle regeneration, and enhancing functional recovery [37, 38]. Similarly, clinical trials involving human subjects have yielded promising outcomes, with hydrogels contributing to pain alleviation and expediting the healing process. Moreover, anecdotal evidence from case studies and real-world applications underscores the practicality and advantages of utilizing hydrogels in the prevention and management of muscle strains among athletes and sports medicine practitioners [39, 40].

**Fig. (3):**
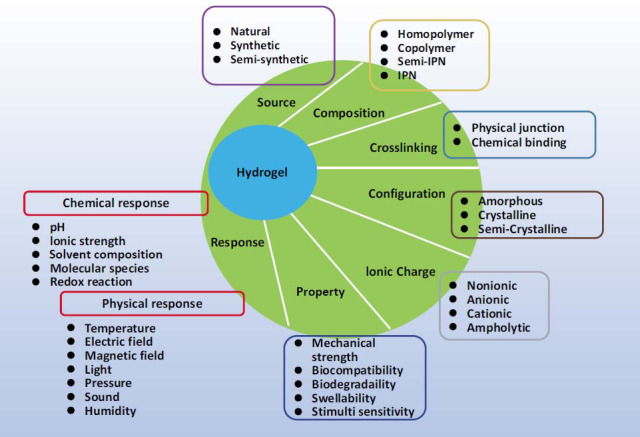
Hydrogels: properties and applications in biomedicine (copyright acquired from Ho *et al.* [41]).

Nevertheless, several hurdles must be surmounted to optimize the utilization of biomedical hydrogels in muscle strain management (Fig. **3**). These challenges encompass the selection of appropriate hydrogel materials possessing desirable attributes, fine-tuning the degradation rate to align with the healing trajectory, augmenting the bioactivity and stability of encapsulated therapeutics, and comprehending the long-term ramifications and potential risks associated with hydrogel administration.

This comprehensive review offers an in-depth analysis of current research pertaining to biomedical hydrogels for managing EIMD. Key focal points include the challenges inherent in achieving optimal biocompatibility and replicating the mechanical attributes of native muscle tissues. Additionally, the review delves into advancements in controlled drug release systems embedded within hydrogels, with a particular emphasis on strategies aimed at attaining precise control over drug release kinetics. Furthermore, it explores the integration of bioactive molecules, growth factors, and stem cells into hydrogel formulations to bolster their regenerative capacity. Lastly, the review underscores the significance of clinical translation, long-term efficacy, and prospects in this evolving field.

## 2 CHARACTERISTICS AND PROPERTIES OF BIOMEDICAL HYDROGELS

Biomedical hydrogels, defined as three-dimensional crosslinked networks of hydrophilic polymers, are composed of either natural or synthetic materials [41, 42]. Natural constituents include collagen, gelatin, hyaluronic acid, chitosan, and other polysaccharides and fibrin, while synthetic materials predominantly consist of hydrophilic polymer [43]. Collagen and alginate are also viable raw materials for hydrogel preparation [44]. These hydrogels have attracted significant attention in the field of biomedicine [41, 42]. Notably, their high water content, comparable to that of natural biological tissues, enables them to closely mimic the extracellular matrix environment. In the context of managing EIMD, biomedical hydrogels offer a wide range of properties and advantages, making them highly suitable for therapeutic applications.

### 2.1 High Biocompatibility

A fundamental characteristic of biomedical hydrogels resides in their exceptional biocompatibility, a trait of paramount importance in therapeutic applications. Their resemblance to the ECM facilitates harmonious interactions with cells and tissues, thereby fostering crucial cellular processes such as adhesion, proliferation, and migration [45-47]. This inherent biocompatibility substantially diminishes the likelihood of adverse reactions, rendering hydrogels well-suited for *in vivo* implantation or application.

Furthermore, biomedical hydrogels can be meticulously engineered to exhibit mechanical properties closely mirroring those of native tissues, thereby affording optimal support and seamless integration at the site of injury [48]. An additional salient property of these hydrogels is their remarkable capacity to absorb and retain significant quantities of water. This attribute proves particularly advantageous in the management of exercise-induced muscle strains, as it facilitates hydration of the injured site and sustains an environment conducive to tissue healing [46]. Moreover, the high water content endows hydrogels with exceptional flexibility and elasticity, enabling them to adapt to the movements and stretching of surrounding tissues without impeding mobility or causing discomfort.

### 2.2 Controlled Drug Release Capabilities

Biomedical hydrogels offer the remarkable capacity to be precisely tailored to exhibit controlled drug release capabilities, thereby heralding a promising avenue for therapeutic innovation. By incorporating therapeutic agents such as analgesics, anti-inflammatory drugs, growth factors, or even stem cells, hydrogels serve as efficacious vehicles for delivering these substances directly to the site of injured muscle tissue [31]. This localized drug delivery paradigm presents numerous advantages over systemic administration, including reduced dosage requirements, mitigation of systemic side effects, and enhanced targeted efficacy.

Crucially, the release kinetics of the encapsulated therapeutics can be finely tuned by modulating the composition, structure, and degradation properties of the hydrogel, thereby ensuring sustained release over a desired temporal window [36]. Moreover, the inherent tunability of biomedical hydrogels emerges as a high asset in therapeutic customization. Through judicious modifications in their composition, crosslinking density, and structural configuration, hydrogels can be meticulously engineered to encompass specific mechanical, biochemical, and degradation attributes.

This inherent tunability empowers researchers and clinicians with the versatility and flexibility to tailor hydrogel-based interventions to precisely match the unique requirements of diverse muscle strain presentations, spanning from acute injuries to chronic conditions. Such adaptability in manipulating key parameters endows hydrogel-based therapies with an unparalleled capacity to be intricately tailored to accommodate the individualized needs of patients, thereby paving the way for personalized and optimized treatment strategies.

**Fig. (4):**
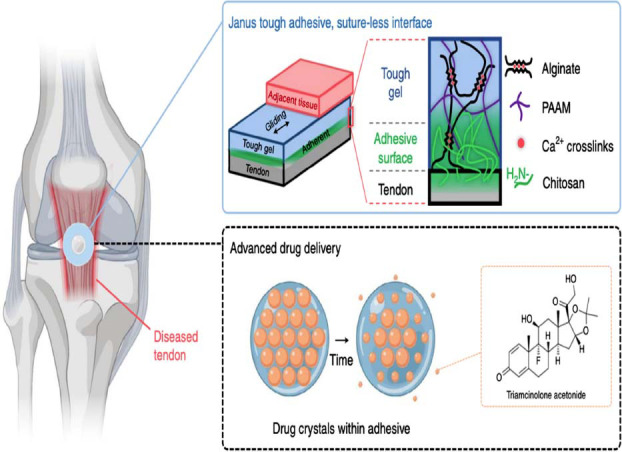
An overview of the vision for the multifunctional performance of the JTAs for tendon (copyright acquired from Freedman *et al.* [57]).

With inherent three-dimensional structure, hydrogels serve as versatile platforms for drug delivery, storage, and release [49]. Traditional administration methods, such as intravenous injection and oral administration, present certain limitations [50]. Intravenous administration achieves rapid peak drug efficacy but requires continuous infusion for sustained effects, which can be burdensome for patients [51]. Oral administration is generally well-received; however, the acidic gastrointestinal environment often compromises drug bioavailability [52]. Hydrogel-based drug delivery system addresses these limitations effectively. The three-dimensional architecture of hydrogels allows for the direct loading of multiple drugs without necessitating chemical modification. Upon placement at the target site, hydrogels can continuously release high concentrations of drugs, preserving drug activity and enhancing therapeutic outcomes [53]. Furthermore, drugs can be chemically linked within the hydrogel matrix, enabling sustained release as the biocompatible hydrogel degrades, thus achieving prolonged therapeutic effects [54]. Additionally, by altering the hydrogel structure, intelligent responsive hydrogels can be engineered to facilitate programmed drug release [55]. These hydrogels respond to changes in pH, temperature, light, electromagnetic fields, or enzymatic activity, adjusting their physical properties and modulating drug release rates to maintain therapeutic drug concentrations [56]. For instance, researchers at Harvard have developed a biocompatible hydrogel known as Janus Tough Adhesive (JTA) (Fig. **4**) [57], capable of high-volume drug loading. Unlike traditional hydrogels, JTA provides robust mechanical support and can release drugs in high doses over short durations without structural failure. In a rat model of Achilles tendon rupture, JTA effectively promoted tendon healing and minimized scar formation. In a rat model of patellar tendon injury, it facilitated the sustained release of the corticosteroid triamcinolone acetonide, reducing inflammation, modulating chemokine secretion, promoting macrophage polarization to the M2 phenotype, and recruiting tendon stem and progenitor cells. Notably, the components of this hydrogel, including alginate, chitosan, and polyacrylamide, are FDA-approved and widely utilized in clinical trials. The potential for localized and on-demand drug delivery positions this hydrogel as a promising candidate for market introduction.

### 2.3 Materials for Tissue Regeneration and Repair

**Fig. (5):**
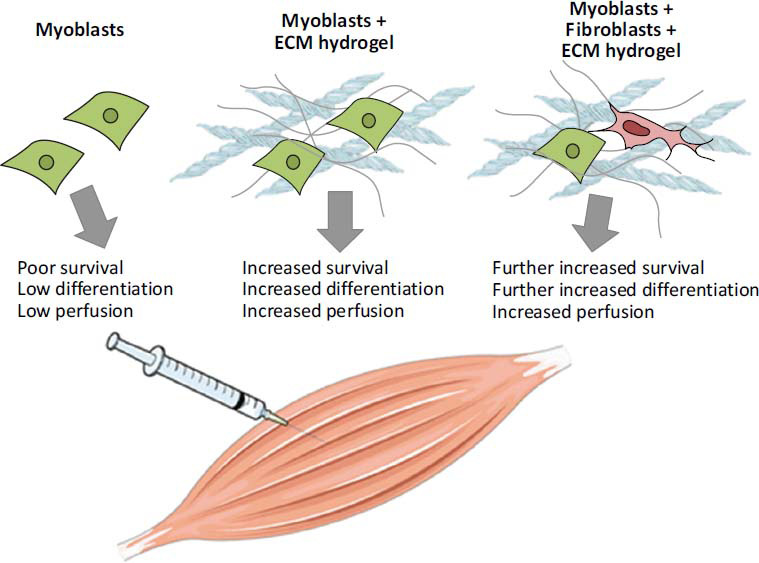
Engineering an injectable muscle-specific microenvironment for improved cell delivery using a nanofibrous extracellular matrix hydrogel. (copyright acquired from Rao *et al.* [58]).

Furthermore, biomedical hydrogels act as promising scaffolding materials for facilitating tissue regeneration and repair, thereby accentuating their multifaceted utility in muscle strain management. By fostering an environment conducive to cell growth and tissue formation, hydrogels play a momentous role in promoting the regeneration of injured muscle fibers and facilitating functional recovery. The intricate three-dimensional architecture of hydrogels serves as a scaffold for facilitating cell infiltration and migration, thereby facilitating the reorganization of tissue architecture (Fig. **5**) [58].

Moreover, the strategic incorporation of bioactive molecules into hydrogel matrices can effectively stimulate crucial cellular activities, including proliferation and differentiation, thereby augmenting the regenerative potential of these materials. In essence, biomedical hydrogels embody a diverse array of properties and benefits that render them highly attractive for managing EIMD. Their exceptional biocompatibility, robust water absorption capacity, controlled drug release capabilities, inherent tunability, and regenerative attributes collectively position them as exemplary candidates for therapeutic applications in muscle strain management.

Continued advancements in research and development within this realm hold considerable promise for the further refinement and optimization of hydrogel-based interventions, thereby facilitating their widespread adoption in the prevention and mitigation of muscle strains stemming from exercise-induced trauma.

## 3 MECHANISMS OF ACTION OF BIOMEDICAL HYDROGELS IN MUSCLE STRAIN MANAGEMENT

Exercise-induced muscle damage hold a prevalent challenge encountered by athletes and individuals actively engaged in physical fitness pursuits. Stemming from heightened or repetitive muscle tension, these strains precipitate tissue damage, inflammatory responses, and associated discomfort. Conventional treatment modalities, while often employed, are encumbered by limitations concerning efficacy and long-term outcomes. Consequently, there arises a compelling imperative to explore innovative approaches, such as biomedical hydrogels, in the realm of muscle strain management [29, 59, 60]. A comprehensive grasp of the underlying mechanisms of action governing the utilization of hydrogels in this context is indispensable for optimizing their therapeutic efficacy.

### 3.1 Providing Enhanced Biomechanical Support

Biomedical hydrogels play a decisive role in muscle strain management through the provision of enhanced biomechanical support, a mechanism underscored by their multifaceted contributions to tissue repair and regeneration [31, 61, 62]. Upon application to the injured muscle, hydrogels function as mechanical reinforcements, adeptly dispersing mechanical loads and mitigating stress concentration on the compromised muscle fibers. This support mechanism effectively attenuates strain on the affected area, thereby forestalling further tissue damage and fostering a conducive environment for healing.

Furthermore, by curtailing excessive movement and imparting stability, hydrogels facilitate the proper alignment of collagen fibers during the healing trajectory. This alignment not only facilitates tissue repair and regeneration but also serves to curtail the formation of excessive scar tissue, thereby optimizing functional outcomes [34, 63-65]. Additionally, the versatility of hydrogels extends to their capacity to serve as reservoirs for therapeutic agents, including analgesics, anti-inflammatory drugs, growth factors, or stem cells, which can be seamlessly integrated into the hydrogel matrix [63]. The controlled release of these therapeutic payloads from the hydrogel matrix enables targeted drug delivery directly to the affected site [34, 64, 65].

This localized drug release paradigm not only enhances therapeutic efficacy but also minimizes systemic side effects commonly associated with conventional drug administration routes. By delivering analgesics, hydrogels afford localized pain relief, thereby alleviating discomfort and augmenting the overall well-being of individuals grappling with muscle strains [66, 67]. Moreover, the release of anti-inflammatory drugs from hydrogels serves to mitigate inflammation at the injury site, a hallmark feature of muscle strains. This anti-inflammatory action not only promotes healing but also expedites the recovery process, thereby culminating in improved clinical outcomes.

### 3.2 Regenerative Properties

The regenerative properties inherent in biomedical hydrogels play a significant role in effectively managing muscle strains. These hydrogels possess the remarkable ability to establish a conducive milieu for tissue regeneration by providing structural reinforcement and facilitating crucial cellular processes such as adhesion, proliferation, and migration [35, 68, 69]. Enabled by their intricate three-dimensional architecture, these hydrogels facilitate the infiltration of cells into the injury site, thereby accelerating the regeneration process of damaged muscle fibers.

Furthermore, biomedical hydrogels can be meticulously engineered to replicate the structural and biochemical attributes of the native ECM, thereby creating a microenvironment conducive to orchestrating essential cellular activities crucial for tissue repair and regeneration. This engineered emulation of ECM characteristics enhances the efficacy of hydrogel-mediated tissue regeneration, fostering optimal healing outcomes in the context of muscle strain management.

### 3.3 Stimulate Cellular Responses

In addition to their structural support and regenerative capabilities, hydrogels can be further augmented by functionalizing them with bioactive molecules or growth factors that elicit specific cellular responses, including proliferation and differentiation. By incorporating these bioactive components, hydrogels stimulate tissue healing and regeneration, thereby enhancing functional recovery following muscle strains. This targeted stimulation of cellular responses shows a crucial characteristic in optimizing the therapeutic potential of biomedical hydrogels in muscle strain management.

Moreover, biomedical hydrogels behave as physical barriers, effectively shielding the injured muscle from external mechanical stressors. This protective function is instrumental in preventing further damage to the already compromised site, thus facilitating uninterrupted healing. Additionally, hydrogels contribute to the maintenance of a moist environment, which is conducive to optimal tissue repair. The high water content inherent in hydrogels ensures that the injured area remains hydrated, thereby facilitating essential cellular processes and supporting the regeneration of healthy muscle tissue [70-72]. This moist environment fosters an ideal milieu for cellular activities, ultimately promoting more efficient tissue repair and functional restoration.

Biomedical hydrogels exert multifaceted therapeutic effects in the management of muscle strains, employing various mechanisms of action to promote tissue repair and functional recovery. Through their enhanced biomechanical support, hydrogels effectively alleviate strain on injured muscle fibers, foster proper tissue alignment, and mitigate excessive scar formation, thereby facilitating an environment conducive to optimal healing [40, 73]. Furthermore, the controlled release of therapeutic agents from hydrogels suggests a leading place in providing localized pain relief, diminishing inflammation, and fostering tissue regeneration. This targeted delivery approach ensures precise administration of therapeutic payloads directly to the affected site, enhancing therapeutic efficacy while minimizing systemic side effects [40, 73]. Through a comprehensive understanding and strategic utilization of these mechanisms, researchers and clinicians can harness the full therapeutic potential of biomedical hydrogels as a promising approach for effectively managing muscle strains induced by exercise.

## 4 APPLICATIONS CONCENTRATION

**Fig. (6):**
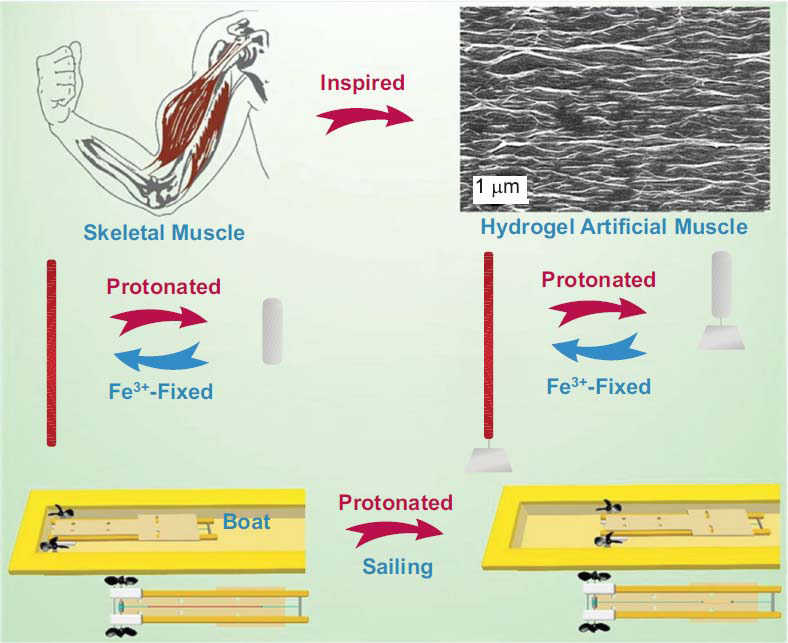
Triple physically cross-linked hydrogel artificial muscles with high-stroke and high-work capacity (copyright acquired from Chen *et al.* [75]).

Exercise-induced muscle damage represent prevalent injuries resulting from the overexertion or repetitive stress endured by muscles during physical exertion. Ranging from mild discomfort to severe damage, these strains manifest with symptoms such as pain, inflammation, and impaired functionality. Effective management of muscle strains is imperative for fostering recovery and reinstating individuals to their desired activity levels. In recent years, biomedical hydrogels have emerged as promising therapeutic modalities for addressing EIMD. Biomedical hydrogels, characterized by their three-dimensional networks of hydrophilic polymers, exhibit a high water content akin to native biological tissues (Fig. **6**) [74, 75]. This intrinsic feature endows them with the capacity to mimic the environment of the ECM, facilitating interactions with cells and tissues [76]. When formulating hydrogels for muscle strain management, numerous properties warrant consideration. These encompass biocompatibility, mechanical characteristics, water absorption capacity, drug release kinetics, tunability, and regenerative potential [76].

### 4.1 Biocompatibility

Biocompatibility represents an important aspect of biomedical hydrogels, ensuring their compatibility with living tissues and precluding adverse reactions upon contact [77]. Hydrogels that closely emulate the ECM exhibit heightened propensity for supporting crucial cellular processes, including adhesion, proliferation, and migration, thereby fostering tissue integration and expedited healing [78]. Moreover, the mechanical properties of hydrogels assume paramount significance, necessitating alignment with those of native muscle tissues to afford optimal support and minimize strain on the injured area [79]. Hydrogels endowed with tunable stiffness and elasticity can seamlessly adapt to the dynamic movements and stretching of surrounding tissues, thereby averting discomfort and preserving mobility [80]. The exceptional water absorption capacity inherent in hydrogels constitutes a strategic advantage in the management of muscle strains, as it facilitates hydration at the injury site and sustains an environment conducive to tissue healing [81]. By absorbing and retaining substantial volumes of water, hydrogels afford sustained hydration, thereby promoting cellular activities essential for tissue repair and regeneration [82]. Furthermore, the capacity to integrate therapeutic agents within hydrogels empowers controlled drug release directly to the injured area. This localized delivery strategy not only mitigates systemic side effects but also optimizes the efficacy of therapeutic compounds, thereby enhancing therapeutic outcomes in the management of muscle strains [82]

### 4.2 Tunability

The tunability inherent in biomedical hydrogels assumes paramount significance, offering a versatile platform for tailoring their properties to cater to diverse types of muscle strains [83]. Through meticulous adjustments in composition, crosslinking density, and degradation characteristics, hydrogels can be customized to precisely align with specific therapeutic requirements [84]. In cases of acute injuries, hydrogels endowed with accelerated degradation rates may be preferred to facilitate prompt initial healing processes. Conversely, for chronic conditions, hydrogels characterized by slower degradation kinetics may offer sustained support, thus promoting long-term therapeutic efficacy [85]. Moreover, the incorporation of bioactive molecules into hydrogel matrices holds promise for augmenting their regenerative potential by eliciting targeted cellular responses, such as proliferation and differentiation. This strategic integration of bioactive components enhances the therapeutic repertoire of hydrogels, fostering enhanced tissue repair and regeneration [86]. These investigations encompass a broad spectrum of facets, ranging from biomechanical support and localized drug delivery to tissue regeneration and functional recovery. Collectively, these studies underscore the immense therapeutic potential of biomedical hydrogels in mitigating the deleterious effects of muscle strains, offering promising avenues for enhanced patient care and rehabilitation.

### 4.3 Other Factors

**Fig. (7):**
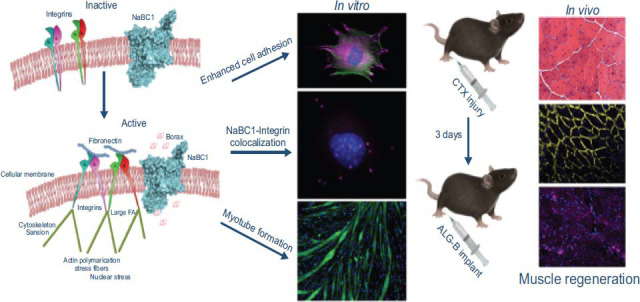
Borax-loaded injectable alginate hydrogels promote muscle regeneration *in vivo* after an injury (copyright acquired from Ciriza *et al.* [91]).

Biomechanical Support: Biomedical hydrogels serve as dominant agents in furnishing enhanced biomechanical support to injured muscles. Acting as a mechanical reinforcement, the hydrogel mitigated stress concentration and strain on injured muscle fibers. This support mechanism facilitated proper tissue alignment, thereby promoting healing processes and enhancing functional recovery [87]. Drug Delivery: The controlled release of therapeutic agents from biomedical hydrogels offers a targeted approach to pain relief and inflammation reduction. Through sustained drug release, the hydrogel effectively mitigated inflammation, alleviated pain, and expedited healing compared to conventional drug administration methods [88]. Tissue Regeneration: Biomedical hydrogels create a conducive environment for tissue regeneration and repair. This hydrogel facilitated the infiltration of regenerative cells, stimulated angiogenesis, and enhanced collagen deposition, thus fostering improved tissue regeneration and functional recovery [89, 90]. Functional Recovery: The regenerative properties inherent in biomedical hydrogels function indispensably in facilitating functional recovery post-muscle strains. The hydrogel facilitated cell infiltration, promoted myofiber regeneration, and bolstered muscle contractile function, underscoring its potential in restoring functionality post-injury (Fig. **7**) [91].

Biomedical hydrogels exhibit considerable promise in the management of EIMD, owing to their unique properties encompassing biocompatibility, mechanical support, controlled drug delivery, regenerative potential, and tunability. These studies underscore the efficacy of biomedical hydrogels in providing biomechanical support, delivering therapeutic agents, promoting tissue regeneration, and facilitating functional recovery. Continued research and development efforts hold the potential to revolutionize the treatment landscape for muscle strains, enhancing patient outcomes and alleviating the burden associated with these injuries.

### 4.4 Smart Hydrogels in Muscle Stretching

With the development of bionics and composite materials, the research of multi-functional intelligent hydrogels has received extensive attention. Through programmed design steps, hydrogels can achieve different structures and functions [92]. Under the premise of ensuring biocompatibility, different natural or synthetic materials are screened, and the structure ratio of materials is adjusted to achieve the construction of composite intelligent hydrogels.

**Fig. (8):**
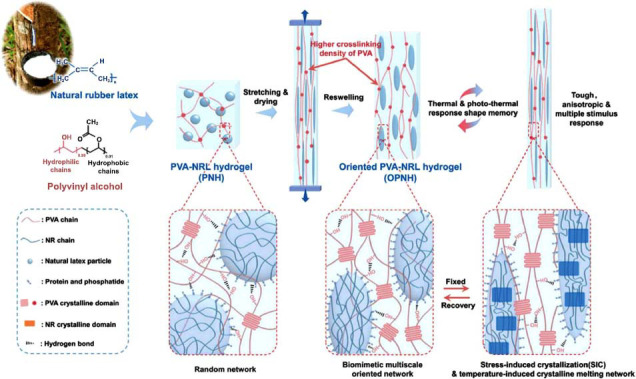
Preparation and micromechanism of muscle bionic multistage oriented PVA/ natural latex composite hydrogel (copyright acquired from Li *et al.* [95]).

Based on the principle of free radical polymerization, multifunctional polyvinyl alcohol (PVA)/carboxymethyl cellulose (CMC) /poly (acrylamide- co-1-vinyl-3-butylimidazolium bromide) (P(AAm- co-VBIMBr))(PCPAV) ionic conductive hydrogels were introduced to act as multi-functional hydrogels with high tensile strength, high elongation at large break, good toughness and strong fatigue resistance. Due to a variety of interactions between copolymers, including covalent crosslinking, multiple hydrogen bond interaction and electrostatic interaction, the obtained ionic conductive hydrogels show excellent function of promoting muscle rehabilitation [93]. To achieve multi-gradient intelligent control and evaluation of finger muscle condition, a research team was inspired by natural materials and aloe vera and prepared a hydrogel with a special structure using skin (named skin-polyvinyl alcohol-polyaniline-AGNWS, S-PPA). S-PPA innovatively uses the hydrogen bond interaction between ions and water molecules to treat the surface of the hydrogel. The S-PPA hydrogel with protective skin has a strong ability to resist damage. Through the rehabilitation system, muscle strength and endurance can be observed in real time to achieve human-computer interaction. Therefore, it has great application potential in the fields of human muscle rehabilitation and biomedical engineering [94]. Muscle bionic hydrogels have also attracted attention. The research team used muscle bionic and microphase composite strategies, took natural latex particles as functional units, and prepared muscle bionic multistage oriented polyvinyl glycol (PVA)/ natural latex composite hydrogels (OPNH) through the reconstruction of PVA crystal network and the properties of natural rubber stretching-induced crystallization (Fig. **8**) [95]. OPNH shows excellent mechanical properties and shape memory response ability, which provides a new shape memory material for the application of intelligent bionic muscles and multi-stimulus response devices. The function of artificial muscles is realized through the contraction and relaxation of smart materials under various stimuli. By introducing dynamic covalent bonds into hydrogels based on cross-linked copolymers of acrylamide and acrylic acid, the team developed a hydrogel material that can respond to environmental stimuli with both reaction speed and force [96]. The material can achieve a fast response to ultraviolet light, and the elastic potential energy stored in the hydrogel can be fully released in a short period of time, exceeding the energy density released by the muscles of real organisms.

## 5 CHALLENGES AND FUTURE PERSPECTIVES

The potential of biomedical hydrogels in exercise-induced muscle strain management is significant, but their widespread application and further advancement require addressing various challenges and opportunities. This section examines key challenges encountered in the field and offers insights into future perspectives concerning the development and utilization of biomedical hydrogels.

### 5.1 Biocompatibility and Immunogenicity Challenges

A prominent challenge in biomedical hydrogel design is attaining optimal biocompatibility to minimize adverse reactions upon contact with living tissues [97]. Despite endeavors to fabricate hydrogels closely mirroring the native ECM, further research is imperative to enhance their biocompatibility, mitigate immune responses, and foster integration with host tissues. Comprehensive comprehension of the interplay between hydrogels and the immune system holds vital importance in surmounting immunogenicity hurdles and refining the biocompatibility of hydrogel materials.

### 5.2 Mechanical Properties and Durability

The mechanical characteristics of biomedical hydrogels are fundamental in furnishing sufficient support and safeguarding injured muscles [98]. Replicating the mechanical attributes of native muscle tissues poses a substantial hurdle [99]. Hydrogels necessitate adequate stiffness, elasticity, and toughness to endure the forces encountered during muscle contraction and relaxation [100]. Furthermore, sustained support throughout the healing trajectory necessitates the long-term durability of hydrogels. The development of hydrogel materials endowed with modifiable mechanical properties and heightened durability will be instrumental in surmounting these challenges.

### 5.3 Controlled Drug Release

Despite offering targeted therapy for pain relief and inflammation reduction, controlled drug release systems integrated into biomedical hydrogels encounter challenges in achieving precise control over drug release kinetics [101]. Designing hydrogels with controlled and programmable drug release profiles holds promise for tailored therapeutic interventions, accommodating the specific stage of muscle strain and individual patient requirements [102]. Further investigation is warranted to refine drug loading strategies, modulate release rates, and engineer stimuli-responsive hydrogels to facilitate on-demand drug release.

### 5.4 Regenerative Potential

Augmenting the regenerative capacity of biomedical hydrogels constitutes a critical facet of their prospective advancement. Integration of bioactive molecules, growth factors, and stem cells into hydrogel formulations holds promise for fostering tissue regeneration and expediting healing processes [103]. Nonetheless, comprehensive research is requisite to discern the optimal combination of bioactive factors and their concentrations to potentiate cellular activities encompassing proliferation, angiogenesis, and myofiber regeneration. Moreover, devising strategies to direct the differentiation of stem cells within hydrogels stands to further amplify their regenerative efficacy.

### 5.5 Clinical Translation and Long-term Outcomes

**Fig. (9):**
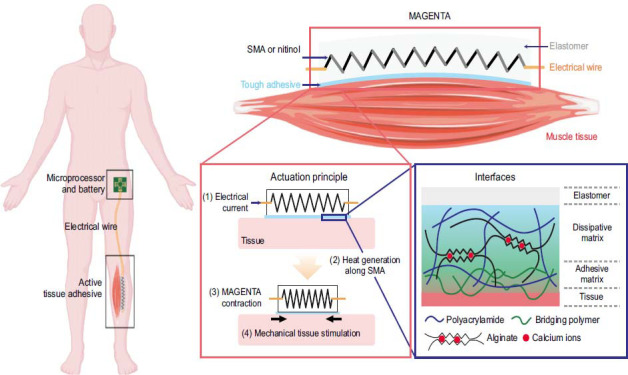
Active tissue adhesive activates mechanosensors and prevents muscle atrophy (copyright acquired from Nam *et al.* [110]).

To facilitate the widespread clinical adoption of biomedical hydrogels, meticulous assessment of their safety, efficacy, and long-term outcomes is imperative [104-106]. Despite promising findings from preclinical investigations, comprehensive and extended clinical trials are indispensable for delineating the effectiveness and safety profiles of hydrogel-based therapies [107, 108]. Furthermore, the standardization of evaluation methodologies, encompassing outcome measures and assessment protocols, assumes paramount importance in enabling cross-study comparisons and establishing evidence-based treatment guidelines [109]. The future trajectory of biomedical hydrogels in the realm of EIMD management brims with potential, warranting focus on several key areas: Advanced Material Design: Continued advancements in materials science, including biofunctionalization techniques and nanotechnology, will enable the development of hydrogels with improved properties and functionalities. Multifunctional Hydrogels: Developing multifunctional hydrogels that combine mechanical support, controlled drug release, and regenerative cues can provide comprehensive solutions for managing muscle strains (Fig. **9**) [110]. Combination Therapies: Synergistic effects from combined interventions can accelerate healing, promote muscle regeneration, and facilitate functional recovery [111]. Patient-Specific Approaches: Personalized medicine approaches, such as patient-specific hydrogel formulations, can optimize treatment outcomes [112]. Commercialization and Accessibility: Expanding the availability and accessibility of hydrogel-based therapies will be crucial for their widespread adoption. Collaboration between academia, industry, and regulatory bodies is needed to navigate manufacturing challenges, establish quality standards, and ensure cost-effectiveness for broad patient access [113]. In conclusion, while challenges exist, the field of biomedical hydrogels for exercise-induced muscle strain management is progressing rapidly. Innovative solutions addressing biocompatibility, mechanical properties, controlled drug release, regenerative potential, and clinical translation are being explored. With continued research and development, biomedical hydrogels hold great promise in improving the treatment outcomes for individuals suffering from muscle strains, enhancing recovery, and reducing the burden associated with these injuries.

## CONCLUSION

Exercise-induced muscle damage is a common injury that can impede athletic performance and daily activities. Biomedical hydrogels present a promising solution for managing these strains, providing support, controlled drug release, and regenerative cues. Despite challenges, ongoing research holds the potential for improving treatment outcomes and reducing strain burdens.

Optimizing biocompatibility and mimicking native muscle tissue mechanics are critical challenges in hydrogel design. Controlled drug release systems in hydrogels offer targeted pain relief and inflammation reduction. Incorporating bioactive molecules, growth factors, and stem cells enhances hydrogel regenerative potential. Rigorous evaluation of safety, efficacy, and long-term outcomes is essential for clinical translation. Collaboration among academia, industry, and regulators is vital for overcoming manufacturing hurdles and ensuring broad patient access.

## LIST OF ABBREVIATIONS

EIMD: Exercise-Induced Muscle Damage
RICE: Rest, Ice, Compression, and Elevation
NSAIDs: Nonsteroidal Anti-Inflammatory Drugs
DOMS: Delayed-Onset Muscle Soreness
RBE: Repeated Bout Effect
CK: Creatine Kinase
ECM: Natural Extracellular Matrix
